# Rhino-Orbital-Cerebral Mucormycosis in a Post-COVID-19 Patient from Peru

**DOI:** 10.1155/2022/2537186

**Published:** 2022-03-15

**Authors:** Linda Ponce-Rosas, Jose Gonzales-Zamora, Nelson Diaz-Reyes, Oliver Alarco-Cadillo, Jorge Alave-Rosas

**Affiliations:** ^1^Peruvian American Medical Society, Especialistas en Gestion de Salud Ocupacional, Lima, Peru; ^2^Department of Medicine, Division of Infectious Diseases, University of Miami Miller School of Medicine, Miami, FL, USA; ^3^Peruvian Union University, Lima, Peru; ^4^Department of Internal Medicine, Good Hope Clinic, Lima, Peru; ^5^Department of Internal Medicine, Good Hope Clinic, Peruvian University of Applied Sciences, Lima, Peru

## Abstract

Mucormycosis has been reported increasingly in patients affected by COVID-19, especially in India where the first cases were described. In Latin America, there is limited information about this association, mainly coming from Brazil, Mexico, and Peru. Herein, we report the case of a 66-year-old female that presented with rhino-orbital-cerebral mucormycosis, diabetic ketoacidosis, and COVID-19. The patient had the compromise of all the sinuses, orbital invasion, and intracranial extension. Isavuconazole was promptly initiated because amphotericin B was not available. She had a single open surgical debridement of necrotic tissues at the beginning of the diagnosis then multiple manual sessions to clear the residual or recurrent disease during approximately 5 months. Isavuconazole was effective and well-tolerated for 10 months without side effects. We highlight the importance of considering mucormycosis in post-COVID-19 patients with uncontrolled diabetes. The report emphasizes the favorable outcome of isavuconazole as an alternative therapy.

## 1. Introduction

Coronavirus disease 2019 (COVID-19) is a disease caused by the novel coronavirus SARS-CoV-2 first documented in China and subsequently causing a lethal pandemic.

According to the World Health Organization (WHO), the pandemic has so far produced more than 318 million confirmed cases and more than 5.5 million deaths throughout the world [1]. COVID-19 has unevenly affected Latin American countries. The Johns Hopkins University database reported that Peru has the highest fatality rate (8.4%) and about 625 people per 100,000 residents have lost their lives to COVID-19 [2].

COVID-19 infection in patients with multiple risk factors such as diabetes mellitus, hypoxemia, and steroid use may increase the susceptibility of opportunistic fungal infections such as mucormycosis (MCR), a disease that can be potentially fatal [3]. Recently, several cases of MCR in people with COVID-19 have been reported worldwide. India, Iran, and Egypt were the most affected countries with hundreds of cases [4–6]. Invasive MCR has been described in patients with severe COVID-19 infection who received corticosteroid and/or tocilizumab as part of their treatment. It has also been associated with mechanical ventilation, supplemental oxygen therapy, and healing rituals with cattle manure. In the majority of patients, diabetes mellitus (DM) was a predisposing condition [4–9]. Similarly, there are increasing numbers of cases reported in Brazil, Mexico, and Peru [7–11]. Here, we report a case of rhino-orbital-cerebral mucormycosis (ROCM) in a patient recovered from COVID-19 infection with uncontrolled DM.

## 2. Case Report

A 66-year-old woman with a 5-year history of poorly controlled diabetes mellitus was admitted to the hospital with drowsiness. Two weeks earlier, the patient went to her primary care physician (PCP) because her son was diagnosed with COVID-19 infection at work. At that time, she was diagnosed with COVID-19 infection by RT-PCR from nasopharyngeal swap. During the evaluation, she reported approximately ten days of headache, for which she self-medicated with dexamethasone 8 mg PO daily for 10 days (a medication that can be obtained without prescription in Peru). Dexamethasone was stopped immediately by her PCP. She had a mild infection with sore throat, headache, fatigue, muscle aches, decreased appetite, anosmia, and dysgeusia. No dyspnea, cough, or fever was reported by the patient. She received symptomatic treatment at home with no oxygen supplementation. Four days prior to admission, the patient noticed a violaceous palate lesion and started to develop accentuated fatigue and confusion. In the following days, her symptoms got progressively worse. On arrival, her temperature was 37°C (98.6 F), blood pressure was 145/75 mmHg, pulse was 123/min, and respiratory rate was 22/min. Oxygen saturation was 97% on room air. On physical examination, she appeared to be in moderate distress with altered mental status. Her mucous membranes were dry. On ophthalmic examination, the left eye (OS) had no light perception (NLP), and there was chemosis and mild proptosis. The direct and consensual light reflexes were absent OS. Fundoscopic exam revealed a normal retina and macula and was notable for pallor and atrophy of the optic disc OS. Visual acuity in the right eye (OD) was 20/100 with corrective lens, unchanged from the previous examination as well as a normal anterior segment and fundoscopic exam. Nasal examination showed necrotic tissue and erosion of the nasal septum, edema, and necrosis on the mucosa of both nasal turbinates and lateral walls of the nasal cavity. A black eschar in the hard palate was also noticed. Lung auscultation revealed diffuse crackles. Neurological examination revealed a patient arousable to painful stimuli and oriented to person, place, and time. The Glasgow score was 13/15. Cranial nerves II–XII were grossly intact. Strength was 5/5 in all extremities. Sensory deficit was not found. Deep tendon reflexes were normal. Coordination was normal. There were no meningeal signs.

**Figure 1:**
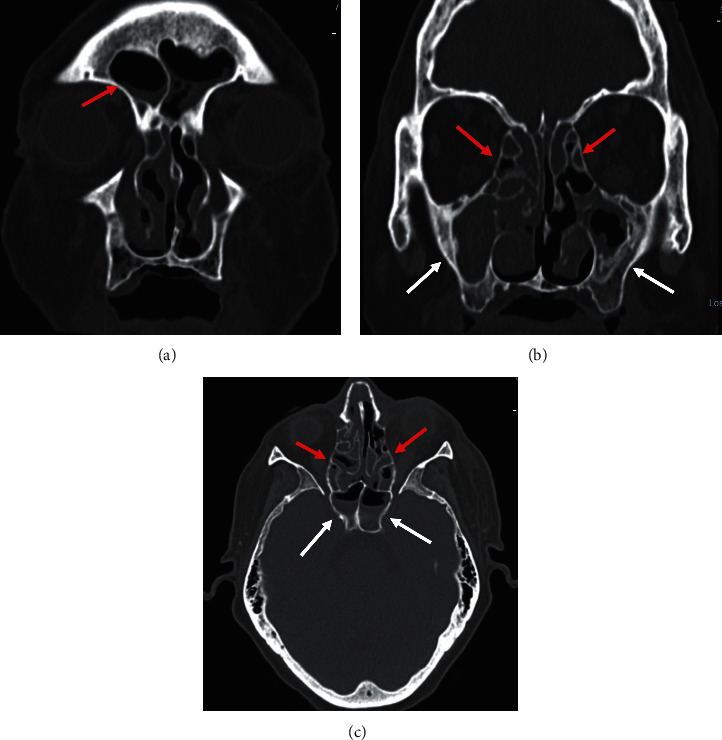
(a) Paranasal sinuses CT scan showed fluid level of frontal sinuses, (b) mucosal thickening of bilateral maxillary (white arrows) and ethmoid sinuses (red arrows), and (c) mucosal thickening of bilateral ethmoid sinuses (red arrows) and fluid level of bilateral sphenoidal sinuses (white arrows).

Laboratory studies on admission showed leukocytosis (white blood cell (WBC) count of 28.5 K/uL with 91% neutrophils and 3.4% lymphocytes). Serum chemistry was significant for hyperglycemia (glucose 407 mg/dL); metabolic acidosis (sodium 135 mEq/L, potassium 3.4 mEq/L, chloride 99.2 mEq/L, and bicarbonate 11.2 mEq/L). Arterial blood gases revealed a pH 7.3, pO2 97.1 mmHg, and pCO2 22.6 mmHg. Hemoglobin A1c was 11%. Creatinine was 0.83 mg/dl. The chest radiograph showed bilateral lung opacities predominantly in the left lower zone compatible with viral pneumonia. Computed tomography (CT) scan showed multifocal small areas of consolidation and perilobular opacities associated with some ground-glass areas in the left lower lobe. CT scan of the brain without contrast was unremarkable. CT scan of paranasal sinuses revealed severe mucosal thickening of the bilateral maxillary sinuses as well as frontal, ethmoid, and sphenoid sinuses. There were no intra, periorbital, or brain lesions (Figure 1). A contrast-enhanced magnetic resonance imaging (MRI) of the brain was not available on admission.

**Figure 2:**
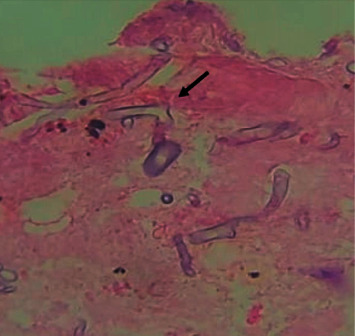
Histopathology showing broad, nonseptate hyphae with 90-degree angle branching (hematoxylin and eosin staining, original magnification ×400).

**Figure 3:**
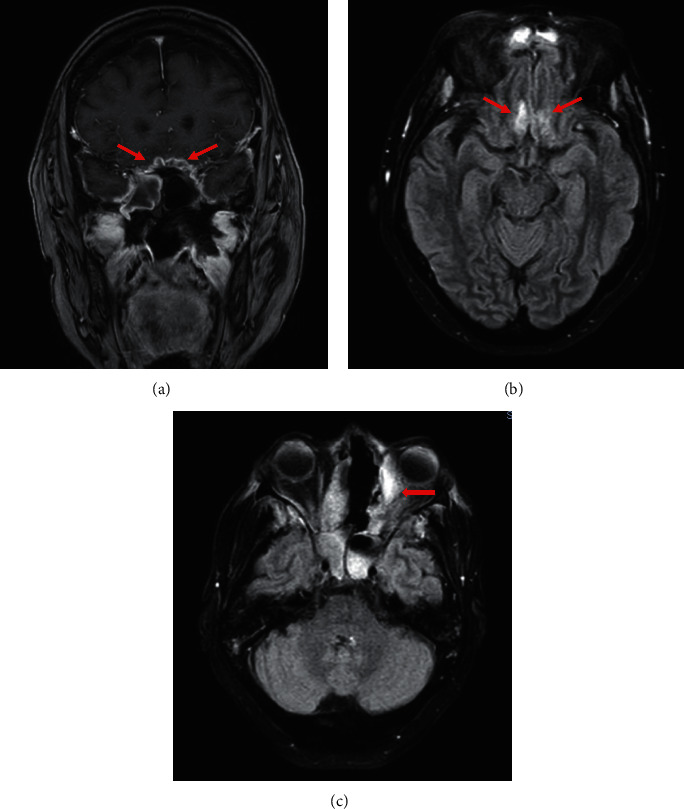
(a) Coronal T1-weighted contrast-enhanced MRI reported frontal anterobasal dural thickening and strong pachymeningeal enhancement (arrows). (b) Fluid attenuated inversion recovery (FLAIR) sequence showed hyperintense signal in cortical anterobasal frontal lobes (arrows). (c) FLAIR sequence revealed an abscess adjacent to the inferior rectus muscle of the left orbit (red arrow).

**Figure 4:**
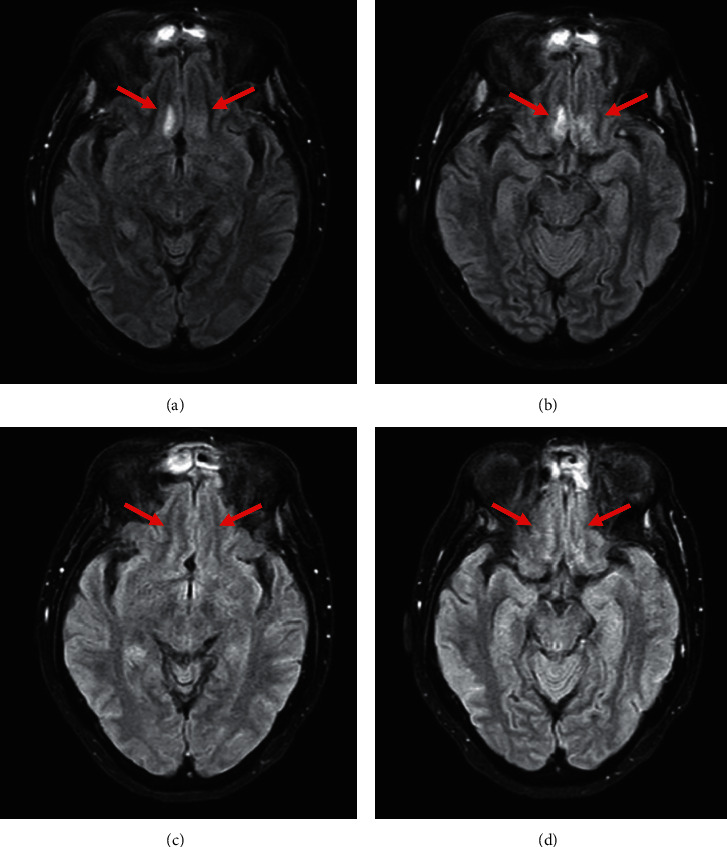
(a, b) FLAIR sequence showed hyperintense signal in cortical anterobasal frontal lobes (arrows) at the beginning of the treatment (first week). (c, d) FLAIR sequence showed reduced hyperintense signal in cortical anterobasal frontal lobes (arrows) at 5 months follow-up.

Due to the development of diabetic ketoacidosis (DKA), the patient was started on intravenous fluids, sodium bicarbonate, and insulin drip. Vancomycin and piperacillin-tazobactam were initiated for suspected bacterial pansinusitis. Isavuconazole (200 mg IV as a loading dose, then 200 mg VO daily) was added for treatment of presumptive invasive fungal infection. Amphotericin B was not available. Maxillofacial surgery was consulted, and the patient was taken to the operating room for an open surgical debridement. Intraoperatively, nasal septum resection was performed, ethmoid sinus antrostomy, and maxillectomy. Left orbital exenteration was indicated but it was not performed due to patient's own choice. Palatal necrosis was managed with palatectomy according to the extension of the invasion. Sinus cultures were positive for multidrug resistant *Pseudomonas aeruginosa*. Vancomycin was discontinued, and piperacillin-tazobactam was switched to colistin and meropenem to complete 21 days. Histopathology revealed broad nonseptated hyphae with right-angle branching consistent with mucormycosis (Figure 2). A contrast-enhanced MRI of the brain was obtained after surgical debridement, revealing orbital invasion and central nervous system (CNS) involvement highly consistent with invasive mycosis (Figure 3). In the following days, the patient improved significantly, showing near resolution of neurologic symptoms and minimal necrotic tissue in affected areas. At 3-month follow-up from admission, she was discharged home with antifungal therapy (isavuconazole) and weekly manual drainage, lavage, and debridement of residual or recurrent necrotic tissues. At 5-month follow-up, the ophthalmic examination revealed exotropia and NLP on the left eye, fundoscopic exam was notable for atrophy of the optic nerve OS. Visual acuity and funduscopic exam were unchanged from the initial examination. At 5- month follow-up, a contrast-enhanced MRI of the brain showed reduced hyperintense signal in cortical anterobasal frontal lobes (Figure 4).

The patient received oral treatment with isavuconazole during 10 months without side effects. She only had a single open surgical debridement at the beginning of the diagnosis then multiple sessions of manual lavage, drainage, and debridement of necrotic tissues for approximately 5 months. Currently, the patient is undergoing periodic ambulatory evaluations to ensure eradication of the disease.

## 3. Discussion

Mucormycosis in patients with COVID-19 infection has been reported worldwide. In a systematic review by Bhattacharyya, the majority of cases (92%) of MCR in patients with COVID-19 reported were from India with 350 cases, followed by 15 cases from Iran, 6 from Egypt, and 11 cases from Turkey [5]. In a cross-sectional study conducted by Pakdel et al. [12], 15 cases from Iran were described. Additionally, Fouad et al. [13] reported 26 cases from Egypt. In our region, 2 cases were from Brazil and 1 case was described in Mexico [7, 8, 10]. In Peru, the literature is scarce, with only four cases reported by Elguera-Falcon and Cumpa-Quiróz [11].

COVID-19 infection may cause persistent lymphopenia in 85% of the affected patients, which may potentially result in an increased risk of opportunistic infections [14, 15]. The primary reason that appears to facilitate fungal spores to germinate in people with COVID-19 is an ideal environment of low oxygen, high glucose, acidic environment, high iron levels, and decreased phagocytic activity of WBC due to immunosuppression (SARS-CoV-2-mediated, steroid-mediated or background comorbidities) coupled with several other risk factors including prolonged hospitalization with or without mechanical ventilation [16].

In a systematic review conducted by John et al. [17], 41 confirmed MCR cases in people with COVID-19 were described, and DM was reported in 93% of cases, while 88% were receiving corticosteroids. Similar data were presented by Singh et al. and Bhattacharyya et al. [4, 5]. Collectively, these results suggest a strong relationship of MCR with diabetes and steroid use in COVID-19 patients, which was also seen in our case.

Direct mycological and histological examinations remain the gold standard for diagnosis. The histopathologic identification of mucormycosis (large, broad nonseptated hyphae with right-angle branching) may provide the only evidence of infection because the culture often yields no growth [3, 18]. A CT scan is crucial for the identification of the bony destruction. Contrast-enhanced MRI provides early detection of meningeal or intraparenchymatous spread and intracranial vascular occlusion [3, 18]. In a prospective study conducted by El-Kholy et al. [19], the most affected sinuses were ethmoid (72.2%) and sphenoid (55.6%) sinuses. Sen et al. [6] reported in the orbit, diffuse involvement predominated in 40% (674 of 1731) followed by involvement of the medial orbit in 27% (469). Orbital apex was involved in 21% (371) patients. In the CNS, the cavernous sinus was most commonly involved in 53% (285 of 539). Bilateral CNS involvement was documented in 5% (133 of 2669) cases with the cavernous sinus being the most common route of spread (70%, 299 of 430).

Treatment of MCR consists of a combination of surgical debridement of involved tissues and antifungal therapy [3]. It remains crucial the appropriate treatment of the underlying medical condition, correction of hypoxia, acidosis, hyperglycemia, electrolyte abnormalities, and a judicious evidence-based use of corticosteroids in patients with COVID-19 [4, 5]. Due to the difficulties in establishing a definitive diagnosis, many patients are treated empirically for MCR because they have risk factors for infection and positive cultures and/or compatible clinical syndromes [3]. Intravenous amphotericin B is the drug of choice for initial therapy. Posaconazole or isavuconazole is used as step-down therapy for patients who have responded to amphotericin B. They can also be used as salvage or alternative therapy for patients who do not respond to or cannot tolerate amphotericin B [18, 20, 21]. Our patient received empiric treatment with isavuconazole because amphotericin B was not available on admission. Isavuconazole was effective and well-tolerated by the patient. She did not develop any side effects, such as gastrointestinal symptoms, confusion, decreased appetite, or QT prolongation. Aggressive surgical debridement of involved necrotic tissues should be considered as soon as the diagnosis of any form of MCR is suspected because these areas are unreachable with antifungals. In the case of rhino-cerebral infection, debridement to remove all necrotic tissue can often be disfiguring, requiring removal of the palate, nasal cartilage, and the orbit [18]. However, more recent experience shows that endoscopic debridement with limited tissue removal can also be attempted [18, 19].

Despite early diagnosis and aggressive combined surgical and medical therapy, the prognosis for recovery from MCR is poor [22]. The overall mortality was around 38.6% in cases with concomitant MCR and COVID-19 infection [4–6, 17, 19]. We believe our patient had a good outcome due to a prompt diagnosis and management.

## 4. Conclusion

This case highlights the presentation of ROCM in patients recovered from COVID-19, which is a complication that should be considered in the differential diagnosis for an early detection and appropriate treatment. We advocate a multidisciplinary approach that combines medical management and surgical debridement of necrotic tissues to achieve favorable outcomes. As shown in this report, isavuconazole may be used as an alternative medication for ROCM in places where the administration of intravenous amphotericin is not feasible.

## Data Availability

Not applicable.
